# Enhancing spatial navigation skills in mild cognitive impairment patients: a usability study of a new version of ANTaging software

**DOI:** 10.3389/fnhum.2023.1310375

**Published:** 2024-01-08

**Authors:** Chiara Stramba-Badiale, Cosimo Tuena, Karine Marie Goulene, Pietro Cipresso, Sandra Morelli, Mirko Rossi, Giuseppe D’Avenio, Marco Stramba-Badiale, Giuseppe Riva

**Affiliations:** ^1^Applied Technology for Neuro-Psychology Lab, IRCCS Istituto Auxologico Italiano, Milan, Italy; ^2^Department of Geriatrics and Cardiovascular Medicine, IRCCS Istituto Auxologico Italiano, Milan, Italy; ^3^Department of Psychology, University of Turin, Turin, Italy; ^4^IRCCS Istituto Auxologico Italiano, Cusano Milanino, Italy; ^5^National Center for Innovative Technologies in Public Health, Istituto Superiore di Sanità, Rome, Italy; ^6^Humane Technology Lab, Università Cattolica del Sacro Cuore, Milan, Italy

**Keywords:** virtual reality, aging, mild cognitive impairment, spatial memory, embodiment, navigation, motor system

## Abstract

**Introduction:**

Mild Cognitive Impairment (MCI) often presents challenges related to spatial navigation and retention of spatial information. Navigating space involves intricate integration of bodily and environmental cues. Spatial memory is dependent on two distinct frame of reference systems for organizing this information: egocentric and allocentric frames of reference. Virtual Reality (VR) has emerged as a promising technology for enhancing spatial navigation skills and spatial memory by facilitating the manipulation of bodily, environmental, and cognitive cues.

**Methods:**

This usability study was based on a fully within-subjects design in which seven MCI patients underwent two kinds of VR conditions: participants were required to complete the ANTaging demo both in Oculus Rift S (*immersive condition*) and in Samsung UHD 4K monitor (*semi-immersive condition*). Participants were seated and they had to use a foot-motion pad to navigate and explore the environment to collect and relocate some objects in the virtual environment. Post-interaction, users provided feedback on their experiences. Additionally, usability, potential side effects, data analysis feasibility, and user preferences with immersive and semi-immersive technologies were assessed through questionnaires.

**Results:**

Results indicated higher usability ratings for the semi-immersive setup, with fewer negative effects reported compared to the immersive counterpart. According to qualitative analyses of the interviews, patients do seem to like both VR apparatuses even though the semi-immersive condition was perceived as the most suitable choice because of the size of the screen. Patients generally found it difficult to remember object locations. Participants expressed the need for more practice with the foot-motion pad, despite an overall positive experience. They generally would like to use this system to improve their memory.

**Discussion:**

Identifying these key aspects was crucial for refining the system before the upcoming clinical trial. This study sheds light on the potential of semi-immersive VR in aiding individuals with MCI, paving the way for enhanced spatial navigation interventions.

## 1 Introduction

Mild Cognitive Impairment (MCI) is a term used to describe the transitional stage that occurs between normal aging and the onset of dementia (Petersen, 2011). Amnestic MCI (aMCI) primarily impacts memory function and is often associated with a heightened risk of developing Alzheimer’s disease (AD). In contrast, non-amnestic MCI, which affects cognitive domains other than memory, can potentially lead to various forms of dementia, such as frontotemporal, vascular, or Lewy body dementia (Petersen et al., 2001). Among the cognitive indicators of this stage, age-related impairments in navigation and spatial memory have historically received less attention; however, they are now the subject of growing research interest (Zhong and Moffat, 2018; see Zhong, 2022 for a recent review).

In the process of navigation, it is essential to encode, retain, and retrieve one’s location and path by utilizing cues from the environment (such as landmarks and boundaries) and self-generated motion cues [including motor commands, proprioception, and vestibular information (Lester et al., 2017)].

Spatial memory is dependent on two distinct reference systems for organizing this information: egocentric and allocentric frames of reference (Klatzky, 1998; Burgess, 2008). The egocentric frame of reference is tied to an individual’s position within the environment and is based on the relationships between oneself and surrounding objects. This representational system utilizes the self as a point of reference to establish a representation centered around the body. Conversely, the allocentric frames of reference focus on relationships between objects, regardless of an individual’s location in the environment. In this representational system, objects and/or the environment itself serve as reference points for constructing a representation centered on the world. To navigate the environment effectively, it is essential to adaptively switch between and blend these two spatial references in response to the specific demands of the surroundings (Lester et al., 2017; Zhong and Moffat, 2018). Accordingly, spatial updating can exist through two formats or reference systems in which egocentric and allocentric spatial relations are processed, respectively (Zhong and Kozhevnikov, 2016; Zhong, 2022). On the one hand, egocentric navigation can be defined as a dynamic process in which an individual consistently calculates and updates transient self-to-object relations with surrounding items, landmarks, or locations, while navigating a path relying on internal bodily signals (i.e., proprioception and vestibular feedback) and external signals (i.e., acoustic and optic flow) (Zhong and Kozhevnikov, 2016). On the other hand, survey-based navigation primarily depends on an allocentric reference which is similar to an environmental representation system based on the coordinates of objects, landmarks, and places and their interrelationships (Zhong and Kozhevnikov, 2016).

Topographical disorientation is often considered a significant indicator for diagnosing AD (Serino et al., 2014; Coughlan et al., 2018). Numerous studies have documented deficits in both the allocentric and egocentric frames of reference, as well as difficulties in transitioning between them, in individuals with aMCI (Serino et al., 2014, 2015; Laczó et al., 2015). These observations are corroborated by neuropathological evidence indicating that the highest rate of atrophy has been found in areas of the hippocampus and retrosplenial cortex which are known to be implicated in spatial representations (Vlċek and Laczó, 2014). However, recent research has revealed that problems with navigation and spatial memory can also be observed in other types of neurodegenerative diseases associated with cognitive decline including dementia with Lewy bodies, frontotemporal dementia, Parkinson’s disease, and vascular cognitive impairment (Uc et al., 2007; Nedelska et al., 2017; Tu et al., 2017; Lowry et al., 2020). Notably, subjective complaints about navigation difficulties and deficits have been reported in individuals with MCI regardless of whether it is of the amnestic or non-amnestic subtype (Cerman et al., 2018; Tuena et al., 2021a).

Embodied cognition and embodiment theories hint that cognitive processes are rooted in perception and action (Meteyard et al., 2012). Navigation and spatial memory can be viewed as embodied processes wherein an abstract cognitive representation of space is underpinned by action, perception, and bodily information (Tuena et al., 2021a). Consequently, information from the sensorimotor system and its interactions with the environment plays a significant role in constructing egocentric and allocentric representations of space and recalling this information(Steel et al., 2021). A recent study exploring the mechanisms of embodiment in aging and neurodegenerative diseases proposed that the decline in the sensorimotor system contributes to spatial difficulties (Kuehn et al., 2018).

Virtual Reality (VR) is a suitable technology for assessing and training in navigation and spatial memory, as it amplifies the manipulation of bodily, environmental, and cognitive information (Cogné et al., 2018; Tuena et al., 2021b). It offers multisensory experiences closely resembling the real world, allowing users to interact with their bodies and the virtual environment (Riva et al., 2020). VR can be regarded as an embodied technology (Riva et al., 2019). A recent systematic review emphasized that incorporating bodily cues (i.e., motor commands, proprioception, and vestibular information) during virtual navigation tasks enhances spatial memory (Tuena et al., 2019). Indeed, researchers are currently investigating the potential impact of the bodily information incorporated as part of VR task implementation in terms of both allocentric and egocentric reference frame use (Chrastil and Warren, 2012; Huffman and Ekstrom, 2021; Steel et al., 2021). One way to study and train spatial memory using active navigation is through the ANTaging software (Tuena et al., 2022). This was developed with immersive VR and active navigation principles to train egocentric and allocentric spatial memory in MCI with Cave Automated Virtual Environment (CAVE) (Tuena et al., 2022) and 3dRudder (a foot-motion pad). Active navigation in this VR system involves a high degree of naturalistic motor movements (Tuena et al., 2021b). However, limitations of the previous version of ANTaging were that CAVE technology was not scalable, and expansive technology. Moreover, it was not possible to extract motor data from the foot-motion pad to be studied in parallel with cognitive (i.e., spatial memory) performance.

The objective of this study was to improve ANTaging software and assess two VR low-end (scalable) solutions for future research. We aimed to assess, with both qualitative and quantitative methods, the usability, the side effects, the feasibility of cognitive and motor data analysis, and the acceptance of the newly improved technology in a sample of MCI patients. Inizio modulo.

## 2 Materials and methods

### 2.1 Participants

Seven (four females and three males) patients with MCI syndrome were recruited for the usability study. The mean age was 75 (*SD* = 4.62), the mean year of education was 12.86 (*SD* = 3.89), and the mean of the Mini-Mental State Examination (MMSE) was 28.01 (*SD* = 1.36). MCI diagnosis was carried out by a clinical neuropsychologist and by the physician (CSB, KMG), according to the clinical patient history, neurological referral, and neuropsychological diagnosis based on a comprehensive neuropsychological battery of tests. MCI diagnosis was done according to the core clinical criteria of Albert et al. (2011): (1) concern regarding a change in cognition obtained from the patient, an informant, or a clinician; (2) impairment in one or more cognitive domains (as assessed by clinical neuropsychologist cognitive tests); (3) preservation of independence in functional abilities; and (4) and no dementia diagnosis (as reported by patient history and anamnesis). Additional inclusion criteria were: the absence of severe cognitive deterioration as assessed by the Italian version of the MMSE cut-off score (Magni et al., 1996), age ≥65, and hearing and vision in the normal range or corrected. Exclusion criteria were: (i) the presence of acute stroke/transient ischemic attack that occurred in the 6 months before the visit; (ii) the presence of aphasia and/or neglect; (iii) the presence of other concomitants severe neurological/psychiatric diseases (e.g., hemiparesis, multiple system atrophy, muscular skeletal/orthopedic deficits that limit movement) (iv) presence of physical and/or functional deficits; (v) comorbidity with severe neurological and/or psychiatric diseases (e.g., neoplasms, sclerosis multiple sclerosis, amyotrophic lateral sclerosis, Huntington’s disease, schizophrenia, addiction, personality disorder, eating disorder) or with psychiatric conditions not under drug treatment (anxiety-depressive syndrome, bipolar disorder); (vi) history of head trauma with loss of consciousness and (vii) recurrent vertigo. Previous systematic research has shown that a sample from five to ten is adequate for usability assessment in older people with MCI (Tuena et al., 2020; Contreras-Somoza et al., 2021).

Participants were recruited at the Inpatient Clinic of the Department of Geriatrics and Cardiovascular Medicine, IRCCS Istituto Auxologico Italiano—Mosè Bianchi, Milan. The study was approved by the Ethics Committee (2023_01_31_11) of Istituto Auxologico Italiano and written informed consent was obtained from the participants before they participated in the study.

### 2.2 Equipment

**FIGURE 1:**
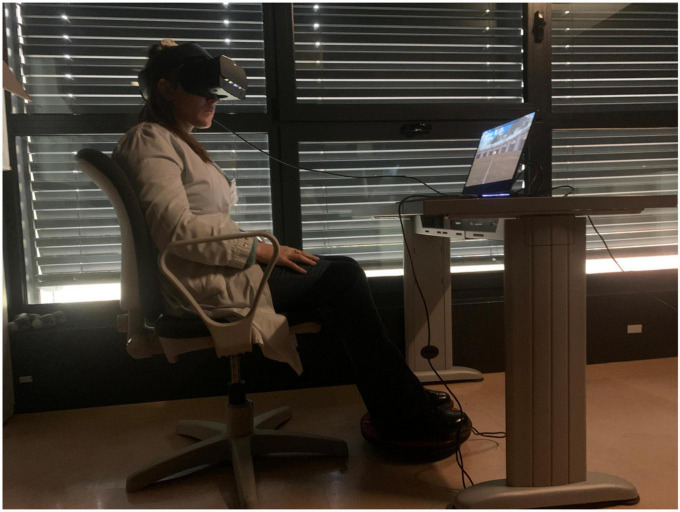
Virtual reality (VR) immersive apparatus. CSB acted as a participant wearing an Oculus Rift S and using the 3D Rudder to navigate through the virtual environment.

**FIGURE 2:**
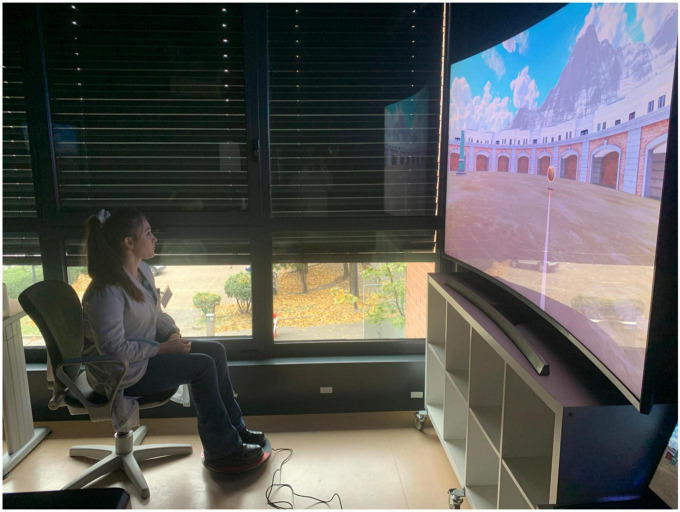
Virtual reality (VR) semi-immersive apparatus. CSB acted as a participant watching a Samsung UHD 4K monitor and using the 3D Rudder to navigate through the virtual environment.

This study was based on a fully within-subjects design in which each participant underwent two kinds of VR conditions: participants were required to complete the ANTaging demo both in Oculus Rift S (*immersive condition*) and in Samsung UHD 4K monitor (*semi-immersive condition*). In both conditions, participants were seated and they had to use the 3dRudder (a foot-motion pad) to navigate and explore the environment. Figures 1, 2 represent the apparatus employed in the study.

The Oculus Rift S is an immersive VR headset that delivers a captivating 3D experience. It achieves this by utilizing a high-resolution display, boasting 1280 × 1440 pixels per eye, ensuring stunning visual clarity. Unlike the CAVE system, it employs inside-out tracking technology, eliminating the need for external sensors or cameras for movement tracking, and simplifying the setup process. The headset also incorporates a comfortable and adjustable head strap, offering a secure fit for users. Integrated speakers provide audio immersion, making it an all-in-one VR solution. The Samsung Ultra High Definition 4K TV is a cutting-edge television that offers an exceptional visual and entertainment experience. It features a stunning 4K UHD display, providing a resolution of 3840 × 2160 pixels. This results in incredibly sharp and lifelike images with vibrant colors and enhanced clarity, allowing to see every detail with remarkable precision. Unlike some older models, this TV is equipped with advanced features such as High Dynamic Range support, which further enhances the contrast and color range for an even more immersive viewing experience.

For this study, a 3dRudder was used to interact with the virtual environment in both conditions. The 3dRudder is a circular platform that is used while sitting in a comfortable and safe position. The patient places the feet on the top to use it. It is, in turn, fixed on a semi-spheric lower section. This solution allows the user to manage the movement intuitively, tilting the feet in the desired direction. Inside the device, inertial sensors and pressure sensors are installed that process the user’s movements and translate them into virtual actions.

### 2.3 Protocol

The usability session lasted approximately 1 h and 15 min, and the participants were asked to complete a demo ANTaging training both with the immersive and semi-immersive conditions. As described in the ANTaging protocol (Tuena et al., 2022), the demo session was composed of encoding and recall phases.

**FIGURE 3:**
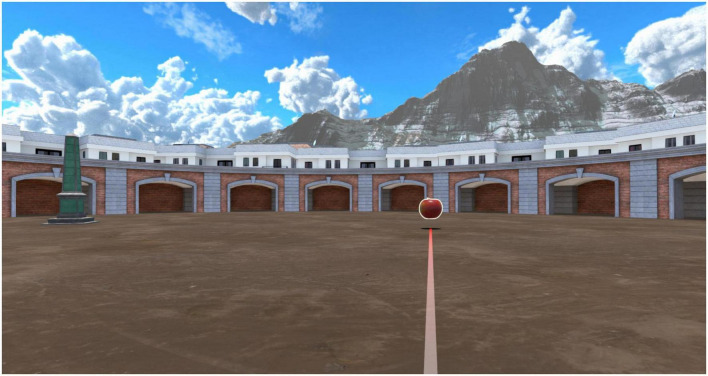
Encoding phase. In the encoding phase, the participants had to follow an illuminated guideline using the 3dRudder and place themselves over each object.

**FIGURE 4:**
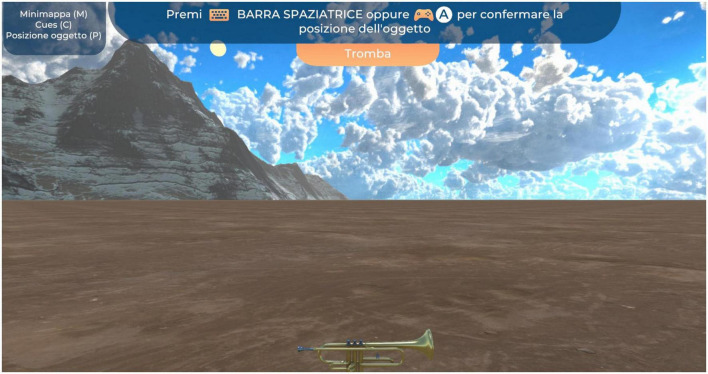
Recall phase. In the recall phase, participants had to recall the object’s exact location.

Figures 3, 4 represent the encoding and recall phases.

The participants received training on how to use the 3dRudder before starting their task. The virtual environment consisted of a circular city square, an obelisk, two mountain ranges, clouds, and an arcade that encircled the square. In the encoding phase, patients used the 3dRudder to reach four items that were presented one at a time, and each object was seen four times in a random order. In the VR environment, they had to follow an illuminated path using the 3dRudder and place themselves over each object, to confirm that they saw it. Each object was associated with a specific sound, to provide feedback the moment they stepped over it. In the recall phase, they had to recall the object’s exact location when they were convinced. The patients located the objects by pressing “A” on the joypad or by pressing the spacebar of the computer which was located next to him/her. As a result, a message was shown: “congratulations” if the position was right (within a radius of 6 or lower virtual units from x and z item coordinates) or “try again” if it was outside that range. This procedure was carried out four times and the obelisk or the arcade was randomly presented to force the use of egocentric or allocentric recall strategies (Tuena et al., 2022). Relaxing music was used as a background sound during the demo. At the end of the demo, the participants were given some questionnaires to complete, aimed at evaluating their user experience.

### 2.4 Measures

The user experiences in the immersive and semi-immersive conditions were evaluated using the following methods.

The System Usability Scale (SUS) (Brooke, 1996) is a “quick and easy to use” questionnaire composed of 10 items in which users need to express the degree of agreement on a five-point Likert scale, from “strongly disagree (1)” to “strongly agree (5)” for each statement (Supplementary Table 1). SUS has proven to be a valuable evaluation tool, being robust and reliable in evaluating a wide range of technologies. The final score can range from 0 “lack of usability” to 100 “optimal usability.” Scores were interpreted according to the 7-point adjective rating scale (Bangor et al., 2009), which is composed of the following levels: “best imaginable (100),” “excellent (85–99),” “good (70–84),” “OK (50–69),” “poor (40–49),” “awful (26–39),” and “worst imaginable (0–25).” The Independent Television Commission-Sense of Presence Inventory [ITC-SOPI; (Lessiter et al., 2001)] is a 44-item, self-report questionnaire that investigates several aspects of the IVR experience (Supplementary Table 2). Participants are required to rate their degree of agreement–disagreement with a five-point Likert scale from “strongly disagree (1)” to “strongly agree (5).” The scoring is obtained by calculating the mean of all completed items contributing to each factor. Specifically, it measures the sense of physical space (SOPS), engagement, ecological validity, and the negative effects of the VR experience. We administered only the negative effects section.

Moreover, a formative evaluation was carried out through a semi-structured interview (Pedroli et al., 2018). The outcome is a description of the main difficulties that emerged during the task and the impact of the problem on the usability study. We administered only the usability and expectations sections.

The usability section is based on three main features: Utilization (effectiveness), Learning (efficiency), and Pleasantness (satisfaction) of the experience.

Table 1 presents the questions included in the semi-structured interview.

**TABLE 1:** Semi-structured interview.

Utilization:
(1) What difficulties did you encounter in carrying out the task?
(2) Was it difficult to use the instrument?
(3) Were there any technical problems during the session?
Learning:
(1) Did you have to ask for help to understand how to use the system?
(2) Did it take you a long time to figure out how the instrument works?
(3) Was the exercise complicated?
Pleasantness:
(1) Did you like the virtual environment?
(2) Were some parts of the system complicated?
(3) Did you have any problems using the 3dRudder and the Oculus Rift S/Samsung UHD 4K monitor?
Expectations:
(1) Would you like to use this system for exercise?
(2) Do you think this system could be useful for other types of patients?

In addition, right after the demo, users were asked (“Which of the two technologies did you prefer?”) to express any preference they found during the interaction, and the responses were analyzed.

### 2.5 Data analysis

Data from the memory task and data obtained from the 3dRudder were analyzed with R (3.6.3 version). Performance data saved in the memory task were actual (encoding) coordinates (x, z) of the tested items, coordinates (x, z) of the recalled (patient’s response) location, time to replace each item at recall, total encoding time, and total recall time.

Regarding the spatial memory recall performance, using Cartesian coordinates of the virtual environment (*x*-and *z*-axis) we computed the Euclidean distance between the actual location at encoding and the recalled location (patient’s response). In addition, we computed polar coordinates obtaining the angle from the encoding location relative to the recalled location. So, we obtained a metric of error/distance (r) and a metric expressed in angle (θ) where the patient’s response was given from each actual encoding item location (Origin). This can be expressed with the two-dimension polar coordinates formula below, where r answers the question “How far is the patient’s response from the actual item location?” and θ to the question “At what angle is the patient’s response from the actual item location?” The former is a metric (r) of distance error from the recalled position to the encoding actual object location, and the latter (θ) is a measure of angular deviation, derived from the polar coordinate system, from the recalled angle to the encoding actual object location.

Regarding the motor data taken from the 3dRudder input device, we saved: *z*-axis rotation (yaw; left-right feet rotations) and *y*-axis rotation (pitch; toe-heel rotations). The former allows to rotate the perspective in the virtual environment to take a specific head direction (left or right), the latter to move forward or backward the perspective (e.g., come closer to an object) by pressing the foot-motion pad with the feet toes or heels. The sampling of this data was 100 Hz (1 sample every 10 ms), which is a suitable sampling rate for human body movements (Godfrey et al., 2008). In addition, encoding and recall time were saved.

We used both raw y and z (only data visualization) data points and vectorized data. For the latter, we employed the method from Schaat et al. (2020) for accelerometer data. We reduced each sample vector to the scalar magnitude value (v_*t*_) with the Euclidean formula below:


$$v_{t}=\sqrt{y_{t}^{2}+z_{t}^{2}}$$

This enabled us to obtain a single metric where higher values determine greater mobility and lower values immobility (e.g., v_*t*_ = 0 the 3dRudder is in a neutral position and so there is no movement in the virtual space). In addition, we computed the Jaccard similarity index (Adamowicz et al., 2020), which enables us to identify the similarity of foot-motion pad movements between encoding and recall. This allows us to show if participants have similar lower limb movements in these two phases and the two virtual conditions (immersive and semi-immersive). The adapted Jaccard formula employed in this case is shown below:

J (v_*t*_ encoding ∩ v_*t*_ recall) = (v_*t*_ encoding ∩ v_*t*_ recall)/ (v_*t*_ encoding ∪ v_*t*_ recall).

A Jaccard similarity index of one represents the exact overlap between encoding and recall; an index of zero stands for no overlap.

Qualitative usability interviews were analyzed using thematic analysis (Clarke and Lane, 2013). This method is usually employed when qualitative data are collected. It allows us to identify the main themes (i.e., patterns in the data) that are important or of interest to address future research. More specifically, at the end of the VR demo, we collected patients’ impressions during the use of VR. We then read and compared all the responses, identifying the most common ones. We organized the answers in a meaningful and systematic manner. Finally, we identified the themes that allowed us to describe and organize the positive and negative aspects of the technological interaction with the software and the devices.

## 3 Results

Five patients diagnosed with MCI syndrome could complete both the immersive and semi-immersive conditions. One patient could not complete the immersive and semi-immersive sessions due to motion-sickness in both conditions, whereas one patient could complete the semi-immersive version but due to cybersickness preferred to interrupt the immersive condition. Another patient perceived cybersickness during the immersive condition but could complete the usability demo. Regarding the SUS score for the immersive session, the average score was 65 (*SD* = 22.97), whereas the average score for the semi-immersive session was 69.17 (*SD* = 25.52). A non-parametric *t*-test revealed that the semi-immersive apparatus was rated as more usable than the immersive version with a statistically significant difference [*t* (6) = −3.28, *p* = 0.017]. Additionally, the Wilcoxon test confirmed these results, with a Wilcoxon W value of 0.00 and a significance level of 0.022.

Table 2 presents the *t-test* scores related to the SUS scale of the immersive and semi-immersive conditions.

**TABLE 2:** *t*-test statistics comparing SUS results in immersive and semi-immersive conditions.

Paired samples *t*-Test					
			Statistic	*df*	*p*
SUS immersive VR	SUS semi-immersive VR	Student’s *t*	−3.28	6.00	0.017
		Wilcoxon W	0.00		0.022

Regarding the item of the ITC-SOPI, the immersive version had a mean value of 2.03 (*SD* = 0.91), whereas the semi-immersive condition had an average score of 1.53 (*SD* = 0.56).

A non-parametric *t*-test revealed that patients reported lower negative effects in the semi-immersive condition compared to the immersive one with a statistically significant difference [*t* (6) = 3.08, *p* = 0.022]. The Wilcoxon test, with a Wilcoxon W value of 15.0 and a significance level of 0.058, also supports these findings, noting that 2 pairs of values were tied.

Table 3 presents the *t*-test scores related to the ITC-SOPI scale of the immersive and semi-immersive conditions.

**TABLE 3:** *t*-test statistics comparing ITC-SOPI results in immersive and semi-immersive conditions.

Paired samples *t*-Test					
			Statistic	*df*	*p*
ITC-SOPI immersive VR	ITC-SOPI NE semi-immersive VR	Student’s *t*	3.08	6.00	0.022
		Wilcoxon W	15.0 ^a^		0.058

^a^ 2 pair(s) of values were tied.

A theoretical thematic analysis (Braun and Clarke, 2006; Maguire and Delahunt, 2017) was conducted to summarize patients’ perceptions while using 3dRudder with the immersive and semi-immersive apparatus.

Table 4 describes the essential themes and impressions of the patients while using the technology and performing the spatial memory task.

**TABLE 4:** Summary of the essential themes while performing the spatial memory task.

Questions concerning the VR task	Patient’s impressions	Themes
(1) What difficulties did you encounter in carrying out the task?	It was difficult to relocate the objects in the correct position. At first, it was a little bit complicated to use the 3dRudder. The chair’s seat should be placed in a higher position. It was difficult to relocate the objects when the perspective changed.	Difficulties in the recall phase.
(2) Was it difficult to use the technology?	No Sometimes it was difficult to understand how to use the 3dRudder.	3dRudder training
(3) Were there technical issues during the session?	No	Technology’s challenges
(4) Did you have to ask for help to understand how to use the system?	No During the recall phase, I had to ask the examiner to repeat the instructions.	Difficulties in the recall phase.
(5) Did it take a long time to understand how the technology works?	No It takes a little bit of practice to understand how the technology works.	Need for training
(6) Was the exercise complicated?	The difficulty concerned the relocation of the objects in the correct position. At first, it was a little bit complicated to understand how to use the technology. Slightly more proficiency is needed in using the 3dRudder. Inizio modulo	Technology’s challenges
(7) Did you like the virtual environment?	Yes, I liked it. No, I don’t like to use the VR since I suffer when using it. Yes, I liked the environment, especially the presence of the mountains.	VR environment preferences
(8) Were some parts of the system complicated?	No It was difficult to relocate the objects in the correct position, especially when the obelisk was not in the environment.	Tasks’ challenges
(9) Did you have problems using the platform the Oculus Rift S/Samsung UHD 4 k monitor and the 3dRudder?	No No, now I feel that I can use VR to train	Competence in using VR
(10) Would you like to use the system for exercising?	Yes No, because I suffer while using VR technology	VR negative effects
(11) Do you think this system could be useful for other types of patients?	Yes Yes, especially if a patient is motivated to train their memory. Yes, if they have some problems with their memory.	Patient’s motivation in memory rehabilitation
(12) Did you prefer to use the Oculus Rift S or the Samsung UHD 4 k monitor?	I preferred the Samsung UHD 4 k monitor as I could see the objects more clearly. Sometimes it was difficult to see properly objects with the Oculus Rift S.	VR preferences

According to qualitative analyses of the interviews, patients do seem to like both VR apparatuses even though the semi-immersive condition was perceived as the most suitable choice because of the size of the screen. Patients generally found it difficult to remember object locations. They think that some practice is required but they generally would like to use this system to improve their memory and they think that it could be useful for other types of patients.

When asked to choose one of the versions tested, 71% of the patients preferred the semi-immersive version, one patient (or 14.3%) preferred the immersive, and one patient suffered cybersickness in both conditions and did not report any preferences. More specifically, participants preferred the semi-immersive condition over the immersive one because they reported that they could watch the objects better due to the screen size and image quality. On the contrary, with the Oculus Rift S, they experienced visual fatigue or cybersickness.

**FIGURE 5:**
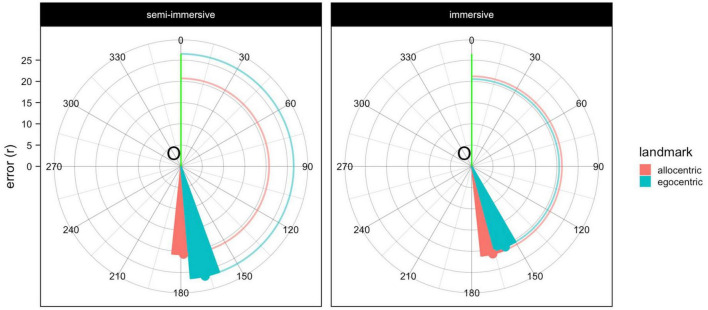
Plot depicting the performance of the spatial memory task. Circular red and blue lines that interpolate the *x*-axis and the triangles show the distance error (r), whereas the angle is represented using the red and blue triangles that point to the angular deviation (θ). The closer the triangles are to the green line the better the angular performance, the closer the lines are to the origin (O) the better the error performance.

Concerning the spatial memory task, Table 5 and Figure 5 summarize the trends of the two conditions. We employed ANOVA with linear mixed-effects model as outlined by Luke (2017). This method does not collapse individual observations as classic ANOVA, consequently, missing trials are not dropped out (Brown, 2021). This resulted in an estimated number of DenDF (rounded 150; i.e., the trials number not the number of participants per se). For example, Table 5 DF computation is based on 160 observations from 5 MCI patients having both immersive and semi-immersive, and two MCI patients having just semi-immersive (i.e., 160 trials). Same methodology was applied for average vt for the Phase and Condition (Table 6). We included the accurate degrees of freedom (DenDF) and ANOVA method specifications in the notes for Tables 5 and 6. There is no significant difference between the conditions and landmarks. The average time to complete the encoding phase (collect four items four times each) in the immersive condition was 10.3 (SD = 3.21) minutes and in the semi-immersive was 8.51 (SD = 2.29) minutes. A non-parametric paired *t*-test showed no significant difference (*p* = 0.375).

**TABLE 5:** Summary of the spatial memory task.

Landmark	Condition	Error (r)	*F*-test	Angle (θ)	*F*-test
Allocentric	Semi-immersive	20.70 (11.32)	Landmark: *F*_(1,150)_ = 1.9, *p* = 0.170	178.32 (85.12)	Landmark: *F*_(1,150)_ = 0.57, *p* = 0.451
Egocentric	Semi-immersive	26.47 (12.85)		167.62 (78.87)	
Allocentric	Immersive	21.19 (10.79)	Condition: *F*_(1,150)_ = 0.38, *p* = 0.541	166.50 (76.82)	Condition: *F*_(1,150)_ = 0.71, *p* = 0.401
Egocentric	Immersive	20.55 (12.18)	Interaction: *F*_(1,150)_ = 2.98, *p* = 0.086	157.40 (84.95)	Interaction: *F*_(1,150)_ = 0.0, *p* = 0.951

The values under parentheses are SD values. Subscripts represent within-subjects/group df values. ANOVA were computed using linear mixed-effects model with participants as random effect (random intercept) Luke (2017).

**TABLE 6:** Values of v_*t*_ for encoding and recall of the immersive and semi-immersive conditions.

Phase	Condition	v_*t*_	*F*-test
Encoding	Immersive	0.34 (0.31)	Phase: *F*_(1,11)_ = 0.18, *p* = 0.676
Encoding	Semi-immersive	0.32 (0.35)	Condition: *F*_(1,13)_ = 0.08, *p* = 0.779
Recall	Immersive	0.29 (0.31)	Interaction: *F*_(1,11)_ = 0.82, *p* = 0.384
Recall	Semi-immersive	0.4 (0.39)	

The values under parentheses are SD values. Subscripts represent within-subjects/group df values. ANOVA were computed using linear mixed-effects model with participants as random effect (random intercept) Luke (2017). vt was the average vt across the trials for each condition.

**FIGURE 6:**
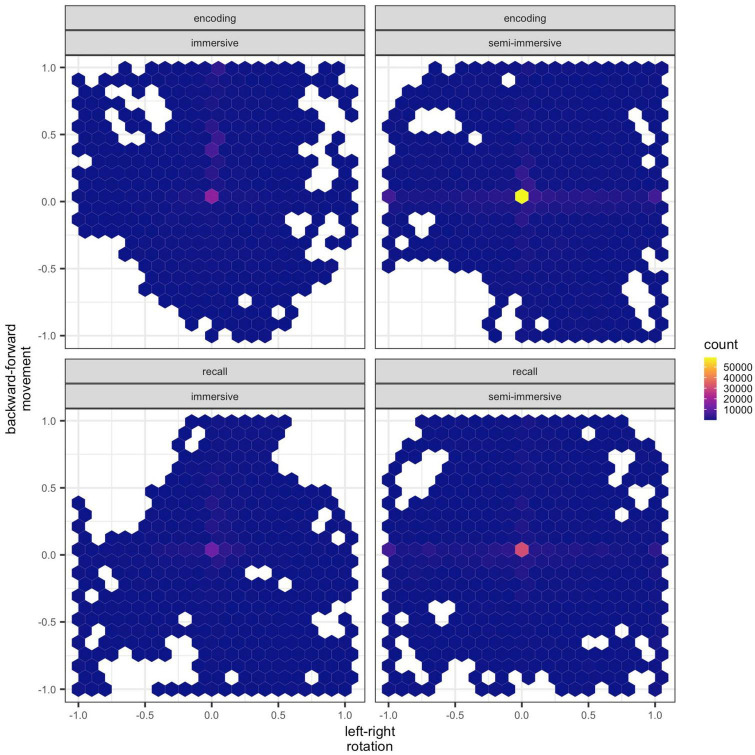
Raw data of the 3dRudder. Hexagons represent clusters of data points. Each hexagon has a width of 0.1 units. Positive *x*-values represent turns to the right, whereas negative *x*-values turn to the left. Positive *y*-values represent forward movement, whereas negative *y*-values backward movements. The lighter color depicts a higher density (count of data points corresponding to the specific coordinates) of data points (y, z) within each hexagon.

Regarding the motor data of the participants who completed the immersive and semi-immersive sessions, the raw data of the foot-motion pad are shown in Figure 6.

The values of v_*t*_ for encoding and recall of the immersive and semi-immersive conditions are shown in Table 6. We found no significant effect. Encoding was similar in the two conditions but recall in the semi-immersive shows greater mobility than the immersive exploration. Concerning the Jaccard similarity index in the two conditions, in the immersive condition the mean index was 0.46 (*SD* = 0.11) and in the semi-immersive condition was 0.33 (*SD* = 0.07). A non-parametric paired *t*-test showed that this difference was not significant (*p* = .375).

Importantly, we report in Table 7 a comparison of SUS and ITC-SOPI negative effects of the old ANTaging CAVE version (Tuena et al., 2023) and the improved prototype we tested here.

**TABLE 7:** Comparison of SUS and ITC-SOPI negative effects of the old ANTaging CAVE version and the improved prototype.

Solution	SUS	ITC-SOPI NE
ANTaging CAVE	60 (15.05)	1.23 (0.31)
ANTaging immersive	65 (22.97)	2.03 (0.91)
ANTaging semi-immersive	69.17 (25.52)	1.53 (0.56)

SUS, system usability scale; ITC-SOPI NE, independent television commission—Sense of presence inventory negative effects. The values under parentheses are SD values.

## 4 Discussion

This study aimed to explore the usability, potential side effects, feasibility of data analysis, and user preferences of the updated ANTaging software when employing immersive and semi-immersive technologies within a sample of individuals with MCI. Both qualitative and quantitative research methods were employed to assess the usability and acceptability of these technologies, which is a critical prerequisite for conducting clinical trials (Contreras-Somoza et al., 2021).

The qualitative aspect of the study involved gathering user comments and feedback. The analysis revealed that most participants found the ANTaging system to be enjoyable but also recommended certain modifications. For example, users encountered visual challenges while completing tasks in the immersive condition, primarily due to certain objects being scarcely visible for their texture. Based on the participants’ response there is a sense that the semi-immersive set-up is the most suitable choice because of the size of the screen which allows them to watch the objects more accurately. Regarding the feedback after the two conditions, we observed that the proficiency in using the technology could benefit from enhancement through practice with the foot-motion pad. In fact, at first, some patients reported some difficulties in navigating employing the 3dRudder. Additionally, users provided generally positive feedback on their overall experience.

As far as the quantitative data are concerned, the SUS analysis underlined that the semi-immersive apparatus was rated as more usable compared to the immersive one as confirmed by the statistical significance. Regarding the item of the ITC-SOPI, patients reported lower negative effects in the semi-immersive condition compared to the immersive one as confirmed by the statistical significance.

Our results were confirmed by recent studies on the usability of a tablet-based application designed for home-based cognitive rehabilitation which revealed a sufficient level of usability (Pedroli et al., 2022).

In the spatial memory task, no substantial significant differences were observed between conditions in terms of errors and angles. However, participants in the egocentric recall condition during semi-immersive navigation showed a higher error rate. The average time to complete the encoding phase was slightly shorter in the semi-immersive condition, though not significantly different from the immersive condition. Both conditions did not differ with respect to either encoding or recall. Greater mobility was evident during the recall phase under the semi-immersive condition compared to the immersive exploration, but this difference was not significant.

The ANTaging semi-immersive condition also improved when compared to the CAVE system (prototype). Similarly to the previous study (Tuena et al., 2023), our findings demonstrated that, beyond graphical elements, older people with MCI place significant importance on psychological factors, notably their perceived confidence in using a new technological device (e.g., 3dRudder). This aligns perfectly with established technology acceptance models within the context of aging (Contreras-Somoza et al., 2021). Theoretical thematic analysis offers a promising approach for gathering user feedback on their technology experience (Braun and Clarke, 2006; Clarke and Lane, 2013). Older adults with cognitive impairment may encounter challenges when using technology (i.e., the presence and assistance of a caregiver or an examiner during the VR experience, the presence of clear instructions and training, and the necessity to practice the use of the 3D Rudder). Therefore, it becomes essential to ascertain whether the technology is user-friendly in achieving therapeutic objectives and if users find it enjoyable (Contreras-Somoza et al., 2021). Our usability study findings primarily revolved around the visual and graphic aspects of the system, including object and sign sizes. Additionally, some patients expressed a lack of confidence in using the 3dRudder, although this can be addressed through practice with the device.

Concerning data analysis, we proposed different methods to compute spatial memory performance.

The polar coordinates (distance and angle between current location and origin) system has been recently proposed as the likely format of location representation (Yousif and Keil, 2021). The polar coordinates included r which is a metric of distance error from the recalled position to the encoding actual object location, and which is a measure of angular deviation, derived from the polar coordinate system, from the recalled angle to the encoding actual object location. Then, in this prototype, we collected accelerometer data from the foot-motion pad to study motor patterns and lower limb mobility. Indeed, 3dRudder could also be used for ankle rehabilitation games (Kanbe et al., 2018).

We recognize certain limitations in our study. Firstly, the small sample size is noteworthy. Nonetheless, it’s worth noting that a previous usability review indicated that 5–10 users are generally sufficient to identify most technological issues (Tuena et al., 2020; Contreras-Somoza et al., 2021). On the other hand, the comprehensive analysis of both qualitative tools and questionnaires can be viewed as a notable strength of our research. This usability study was carried out employing two different VR apparatus: immersive and semi-immersive enabling us to underline MCI patients’ preferences during the VR experience. The 3dRudder device facilitates the experimenter’s ability to safely process users’ motor commands and proprioceptive information from their lower limbs during spatial navigational tasks. However, a drawback of this device is its inability to incorporate whole-body proprioceptive and vestibular information.

Furthermore, it would be interesting to evaluate usability after participants have undergone VR training sessions to determine whether their experience with the technology improves and becomes more user-friendly.

Two patients could not complete the immersive condition due to cybersickness, whereas a patient could not complete the immersive and semi-immersive conditions. Therefore, these data are not available; however, the purpose of this study was to understand which interface was the best to limit the drop-out rate during the trial.

The mean score of 65 on the SUS may not be considered excellent. Nonetheless, it is important to note that this usability study served as a preliminary test conducted on a prototype of the new application, specifically the second version of the system [first version see Tuena et al. (2023)]. We intend to enhance the most critical aspects of the system before embarking on the clinical trial, thereby advancing research in the field.

## Data availability statement

The datasets presented in this study can be found in online repositories. The names of the repository/repositories and accession number(s) can be found below: https://doi.org/10.5281/zenodo.8399594.

## Ethics statement

The studies involving humans were approved by Ethics Committee (2023_01_31_11) of Istituto Auxologico Italiano. The studies were conducted in accordance with the local legislation and institutional requirements. The participants provided their written informed consent to participate in this study. Written informed consent was obtained from the individual(s) for the publication of any potentially identifiable images or data included in this article.

## Author contributions

CS-B: Data curation, Formal analysis, Methodology, Writing – review and editing. CT: Conceptualization, Data curation, Formal analysis, Methodology, Writing – review and editing. KG: Methodology, Writing – review and editing. PC: Funding acquisition, Supervision, Writing – review and editing. SM: Supervision, Writing – review and editing. MR: Supervision, Writing – review and editing. GD’A: Funding acquisition, Supervision, Writing – review and editing. MS-B: Funding acquisition, Supervision, Writing – review and editing. GR: Funding acquisition, Supervision, Writing – review and editing.
