# Supplementary material for: Enhancing spatial navigation skills in mild cognitive impairment patients: a usability study of a new version of ANTaging software
Source: Front Hum Neurosci. 2024 Jan 8;17:1310375. doi: 10.3389/fnhum.2023.1310375 (PMC10801043; doi:10.3389/fnhum.2023.1310375)
Supplement: Supplementary file 1 [file Table_1.DOCX]

***Supplementary Material***

# Supplementary Figures and Tables

## Supplementary Tables

**Table S1. SUS Questionnaire**

| **Please Express your level of agreement on a scale from 1 to 5 regarding the following statements** | Strongly disagree | Partially disagree | neither agree nor disagree | Partially agree | Strongly Agree |
| --- | --- | --- | --- | --- | --- |
| I think that I would like to use this system frequently | 1 | 2 | 3 | 4 | 5 |
| I found the system unnecessarily complex. | 1 | 2 | 3 | 4 | 5 |
| I thought the product was easy to use | 1 | 2 | 3 | 4 | 5 |
| I think that I would need the support of a technical person to be able to use this system | 1 | 2 | 3 | 4 | 5 |
| I found the various functions in the product were well-integrated | 1 | 2 | 3 | 4 | 5 |
| I thought there was too much inconsistency in this system | 1 | 2 | 3 | 4 | 5 |
| I imagine that most people would learn to use this system very quickly | 1 | 2 | 3 | 4 | 5 |
| I found the system very awkward to use | 1 | 2 | 3 | 4 | 5 |
| I felt very confident using this product | 1 | 2 | 3 | 4 | 5 |
| I needed to learn a lot of things before I could get going with this system | 1 | 2 | 3 | 4 | 5 |

**Table S2. ITC-SOPI Negative Effects**

| **Please Express your level of agreement on a scale from 1 to 5 regarding the following statements** | Strongly disagree | Partially disagree | neither agree nor disagree | Partially agree | Strongly Agree |
| --- | --- | --- | --- | --- | --- |
| After my experience of the displayed environment, I felt disoriented | 1 | 2 | 3 | 4 | 5 |
| During my experience of the displayed environment, I felt tired | 1 | 2 | 3 | 4 | 5 |
| During my experience of the displayed environment, I felt visual fatigue | 1 | 2 | 3 | 4 | 5 |
| During my experience of the displayed environment, I felt nausea | 1 | 2 | 3 | 4 | 5 |
| During my experience of the displayed environment, I experienced a headache | 1 | 2 | 3 | 4 | 5 |

**
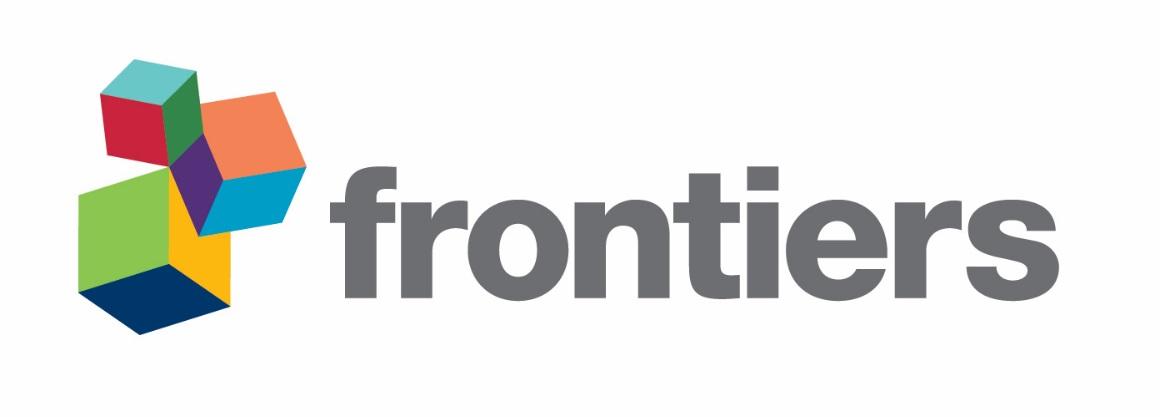
**
